# Problems after flight: understanding and comparing Syrians’ perspectives in the Middle East and Europe

**DOI:** 10.1186/s12889-021-10498-1

**Published:** 2021-04-13

**Authors:** Andrea Drescher, Nikolai Kiselev, Aemal Akhtar, Ceren Acarturk, Richard A. Bryant, Zeynep Ilkkursun, Roland von Känel, Kenneth E. Miller, Monique C. Pfaltz, Matthis Schick, Ulrich Schnyder, Marit Sijbrandij, Julia Spaaij, Naser Morina

**Affiliations:** 1 Department of Consultation-Liaison Psychiatry and Psychosomatic Medicine, University Hospital Zurich (USZ), University of Zurich (UZH), Culmannstrasse 8, 8091 Zurich, Switzerland; 2 Medical Faculty, University of Zurich (UZH), Zurich, Switzerland; 3 School of Psychology, University of New South Wales, Sydney, NSW 2052 Australia; 4 Department of Clinical, Neuro- and Developmental Psychology and WHO Collaborating Centre for Research and Dissemination of Psychological Interventions, Vrije Universiteit Amsterdam, Amsterdam, The Netherlands; 5 Department of Psychology, Koc University, Istanbul, Turkey; 6 War Child Holland, Hemholtzstraat 61, 1098 LE Amsterdam, The Netherlands

**Keywords:** Refugees, Syrian refugees, Mental health, Self-reported problems, PSYCHLOPS, Client-generated outcome measure, Post-migration stressors, Multi-country

## Abstract

**Background:**

Syrian refugees and asylum seekers (SRAs) face multiple stressors after flight, which may vary due to different geographic, economic, cultural and socio-political contexts in the host countries. Past research has recognised the importance of participants’ own perspectives. The aims of this multi-country study were to identify and compare self-reported problems of SRAs between various settings.

**Methods:**

A semi-structured client-generated outcome measurement was used to collect data among adult SRAs in Jordan (*N* = 61), Turkey (*N* = 46) and Switzerland (*N* = 57) between September 2018 and November 2019. Answers were analysed following thematic analysis.

**Results:**

Over half of the participants reported practical problems with an emphasis on camp-related problems (Jordan), finances (Turkey), employment (Jordan and Switzerland) and government regulations (Switzerland), followed by psychological, and social issues.

**Conclusion:**

This study highlights the impact of local contextual factors on wellbeing. The findings emphasise that planning preventative procedures and mental health care services for SRAs need to consider local challenges affecting the population in specific countries.

**Supplementary Information:**

The online version contains supplementary material available at 10.1186/s12889-021-10498-1.

## Introduction

The Syrian civil war has resulted in Syrians being the largest forcibly displaced population over recent years. More than 13 million people have left their homes with 6.1 million internally displaced people, 6.6 million refugees and 118,445 asylum-seekers by the end of 2019. They are hosted in 127 countries, with 83% having fled to neighbouring countries: Turkey hosts most Syrian refugees with 3.6 million, Lebanon hosts 910,600, and Jordan hosts 654,700 [1]. A vast number of Syrian refugees and asylum seekers (SRAs) also arrived in Europe, including Germany having registered 572,800 [1] SRAs and Switzerland almost 20,000 by the end of 2019 [2]. Forcibly displaced people are frequently exposed to potentially traumatic events and other stressful experiences in their countries of origin, as well as during and after flight, which may impact their mental health and overall functioning [3, 4]. Past research has emphasised the importance of a combination of pre- and post-migration stressors, whereby the post-migration environment can play a significant role in modulating recovery from war-related trauma and grief [5, 6]. Displacement-related stressors include, but are not limited to (1) poverty, (2) uncertainty of asylum seeking process, (3) unemployment/dependency on aid, (4) family conflict, and (5) loss of social network [6–8].

These stressors impact mental health directly or through their adverse impact on the social ecology, which refers to one’s relation with the social and family environment [6, 9]. It is argued that each host country presents a unique set of post-migration factors, which partly results from local policies. Therefore, the type and severity of post-migration stressors can differ widely between countries [10]. Previous studies in Jordan’s camps have reported displacement-related problems such as poor living conditions, insufficient access to services such as medical, sanitation [11], marginalisation from international and regional agencies, socio-economic pressures [12] and work exploitation [13]. Concerning the legal status, in Turkey, Syrians are under “temporary protection” meaning they can stay as “guests” until they reside in a third country [8]. There are also thousands of Syrians without temporary protection status and an unknown number of Syrian irregular migrants [14]. Similarly in Switzerland, from over 20′000 Syrian refugees and asylum seekers by the end of 2019, almost half of them hold only the “N” (asylum seeker) or “F” (temporarily admitted refugee) permit meaning a tolerated stay, not the recognition as refugees in Switzerland [2, 15]. These permits need to be renewed every 6 (N) or 12 (F) months [16]. ‘N’ permit holders have serious restrictions in accessing the labour market, governmental subsidies, leaving the country and no right to family reunion [17]. It applies to both country that the temporary protection status leads to feelings of uncertainty and fears of being sent home [8, 18]. Concerning employment in Turkey, since the “Regulation on Work Permit on Refugees under Temporary Protection” in January 2016, access to formal employment has been established [19]. However, the issue of work permits is limited by specific criteria, and illegal work as well as unfavourable working conditions are widespread. Unsurprisingly, employment problems and poverty are reported as most salient problems among Syrians in Istanbul, Turkey [8]. Work permit challenges based on the legal status were also significant problems for Syrians in Switzerland [18]. In Turkey, Syrians have established their own neighbourhoods and communities in Istanbul and cities along the border due to the vast influx. Despite commonalities between Syria and Turkey such as cultural and geographical proximity, increased discrimination towards Syrians due to their nationality, culture, and language has been reported in Turkey [14]. Discrimination, feelings of marginalisation and language difficulties related to entitlements based on legal status such as language courses have been reported by previous research in Switzerland [18]. Stressors and mental health of SRAs have been predominantly assessed through nomothetic measurements - often developed and validated in western contexts [20]. Studies have shown that standard instruments often fail to encompass all aspects of distress in cross-cultural settings, and that it is a fallacy to assume that symptoms in different cultural contexts carry the same significance across cultures [21–23]. Client-generated outcome measurements are person-centred by definition and unlike standardized measures, they seek to encompass the individuals’ phenomenological experiences as well as their priorities, local values, perspectives and expectations [22, 24, 25]. Individuals’ social circumstances and culture are at the core of the WHO global strategy on person-centred care, which suggests that care should address physical, socioeconomic, mental and emotional wellbeing [26]. A person-centred approach is appropriate for understanding the stressors and needs of SRAs because they emigrate to diverse cultural, social, political and religious contexts. Furthermore, despite general cultural commonalities among SRAs, there is also great diversity among Syrians and their individual experiences [21, 27]. This approach also accords with the UNHCR mental health and psychosocial support (MHPSS) programs. These have highlighted the need for a culturally safe environment and respect of diversity when exploring Syrians’ perspectives, and hence favour a person-centred approach to psychosocial support and clinical interaction [22].

Moreover, understanding stressors and needs, including non-psychological or social needs, is a crucial first step before the provision of psychosocial support and effective mental health services, or referral to other relevant services [27–29]. Inadequate assessment of post-migration stressors could risk overlooking key sources of ongoing stress in the social ecology that could be targeted for change [6].

Regarding SRAs’ mental health stressors and needs, many assessments have been conducted in Syria’s neighbouring countries, such as Jordan, Lebanon and Turkey [30, 31], and generalizability to Western European countries is questionable. Comparative studies between low- and middle-, and high-income countries may shed light on key environmental factors affecting adjustment needs of SRAs in these different settings. However, such comparative studies are scant [8, 32] and little is known about the SRAs’ salient problems from their own perspective. The overall aim of this study was to use a person-centred approach to investigate self-reported problems of SRAs and to identify key stressors within Jordan, Turkey and Switzerland. A mixed-method study approach was used to gain a detailed understanding of the SRAs’ emic perspectives on their problems. Aside from understanding their emic perspectives, we focus on the similarities and differences between the countries and describe local environmental factors, which might account for the variability.

## Methods

### Setting

Data were collected as part of three studies conducted in Jordan, Turkey and Switzerland which aimed to assess the feasibility of conducting large controlled trials designed to evaluate the effectiveness and implementation of Problem Management Plus (PM+) with SRAs [33–35]. PM+ is a scalable low-intensity intervention developed by the WHO for people affected by adversity aiming to reduce symptoms of common mental disorders [36, 37]. These trials are part of the larger STRENGTHS project “Scaling up psychological intervention for Syrian refugees” [38].

### Participants

The inclusion criteria for participation were: (1) SRA, (2) at least 18 years of age, (3) showing increased psychological distress measured by means of Kessler Psychological Distress Scale (K10) > 15 [39] and reduced psychological functioning measured by the short form WHO Disability Assessment Schedule (WHODAS 2.0) > 16 [40]. Participants were excluded if they had (1) cognitive or neurological impairments, (2) acute medical conditions, (3) severe mental disorders (e.g. schizophrenia), or (4) were at imminent risk of suicide. Additionally, in Turkey non-registered refugees were excluded, as were participants under tutelage in Switzerland.

### Recruitment

In Jordan, participants were recruited through door-to-door-screening from the Village 2 and the highly restricted, fenced off Village 5 in Al-Azraq Refugee Camp. Unlike refugees in other parts of the camp such as Village 2, those in Village 5 were not allowed to leave the area, visit relatives in other parts of the camp, obtain temporary leave permits or access work outside the camp [41]. In Turkey, participants were recruited from Sultanbeyli, an economically disadvantaged neighbourhood in Istanbul via phone calls using the contact list from the collaborating Refugee and Asylum Seekers Assistance and Solidarity Association (RASASA), as well as through direct referral from RASASA social workers*.* In Switzerland, participants were recruited through social media, brochures, word-of-mouth propaganda, and local stakeholders from German speaking cantons.

### PSYCHLOPS measure

Data were collected through the brief and idiographic questionnaire Psychological Outcomes Profile (PSYCHLOPS) based on Ashworth’s original version [42]. At the core of the questionnaire are three domains (problem description, functioning and wellbeing). The participant was asked to give short free-text responses for the following problem items and functioning domain:

1. *“Choose the problem that troubles you most”;*
2. *“Choose another problem that troubles you”; and*
3. *“Choose one thing that is hard to do because of your problem(s)”.*

PSYCHLOPS has been used within large trials designed to test the effectiveness of PM+ in communities affected by adversity in Kenya [43] and Pakistan [44].

### Data collection

PSYCHLOPS data were collected between the end of January and beginning of February 2019 in Jordan, in September 2018 in Turkey and between January and November 2019 in Switzerland. Research assistants were native Arabic speakers and received a one-day (Jordan, Turkey) or three-day (Switzerland) training in data collection methods and research ethics. After explaining the study by a research assistant to the participant who had previously screened positively, written informed consent was obtained. Gender-matching with participants was achieved in most cases. In order to access illiterate participants, in Jordan and Turkey PSYCHLOPS was conducted as a guided interview measure, whereas in Switzerland this was done only if necessary. Assessments were conducted in confidential rooms to ensure privacy using paper-pencil method in Jordan, in separate rooms in the building of RASASA outside official hours using paper-pencil method in Turkey, and in separate rooms of specialized centres on computer using the Multi Adaptive Psychological Software [45] in Switzerland. PSYCHLOPS responses were recorded by the participants (Switzerland) and research assistants (Jordan, Turkey, Switzerland) in one or two short sentences to encapsulate the participants’ problems as closely and concise as possible.

### Data analysis

Analysis of SRAs’ self-reported problems focused on the first two questions of the PSYCHLOPS tool (describing experienced problems). Translations of Jordan’s and a number of Switzerland’s responses were conducted by a mental health professional interpreter. Responses in Turkey were translated by a bilingual research member.

For analysis we used a data-driven approach in order to respect the geographical and cultural diversity and employed thematic analysis according to Braun & Clarke [46]. Focusing on the semantic meaning, all problem items were given a code. Codes were each compared and similar themes were condensed per question and country. These themes were integrated into broader categories, again compared, and refined to minimize overlap and maximize coherency and consistency. The research team then reviewed and mutually agreed on the coding framework. Finally, a psychology student and AD coded the data set separately according to the final coding framework. The interrater reliability was k = 0.98 for all three countries. Once the coding was completed, similarities and differences across the three countries were investigated. The coding process and the reliability calculations were performed using NVivo 12 software [47].

The socio-demographic variables were statistically analysed using SPSS Statistics 25 [48]. The variable “Age” was normally distributed. Years since flight from hometown, from Syria and arrival in host country were not normally distributed. Therefore, for these variables median (*Mdn)* and inter-quartile range (Q) will be reported.

## Results

### Demographic characteristics

Overall, 164 participants participated across the three trials (Jordan = 61, Turkey = 46, Switzerland = 57). Table 1 shows the socio-demographic characteristics across the three study sites. The main differences were that the participants in Switzerland left their hometown 2 years earlier, were considerably more educated, more frequently divorced/separated or unmarried and more students than participants in Jordan and Turkey. Despite the high number of working permits in Switzerland, the unemployment rate was the highest in Switzerland and more participants in Jordan and Turkey pursued gainful employment.

**Table 1:** Demographic characteristics of the three study populations

Variable	Jordan	Turkey	Switzerland	Total
Total number of participants (n)	61	46	57	164
Mean age (SD), years	43 (8.5)	38 (10.9)	40 (10.0)	40 (9.9)
Age range [min-max], years	22–68	19–61	21–63	19–68
Years since	*Mdn* [Q1,Q3]	*Mdn* [Q1,Q3]	*Mdn* [Q1,Q3]	*Mdn* [Q1,Q3]
having left hometown	5 [4,7]	5 [4,6]	7 [5,8]	6 [4,7]
having left Syria	5 [3,6]	4 [2,4.75]	6 [5,7]	5 [3,6]
having arrived at host country	4 [3,6]	3 [2,4]	4 [1.75,5]	4 [3,5]
	n [%N_J_]	n [%N_T_]	n [%N_S_]	N [%N]
Gender, female	39 [64]	31 [67]	29 [51]	99 [60]
Living situation				
camp/asylum seeker centre	61 [100]	0 [0]	12 [21]	73 [45]
community	n/a	46 [100]	38 [67]	84 [51]
other	n/a	n/a	6 [11]	6 [4]
Education				
no education	13 [21]	3 [7]	0 [0]	16 [10]
basic education	39 [64]	29 [63]	25 [44]	93 [57]
secondary education/technical certificate	8 [13]	7 [15]	21 [37]	36 [22]
tertiary education	1 [2]	7 [15]	11 [19]	19 [12]
Education not completed	17 [33]	25 [54]	40 [70]	77 [47]
Marital status				
married/cohabiting	56 [92]	37 [80]	41 [72]	134 [82]
divorced/separated	2 [3]	2 [4]	6 [11]	10 [6]
never married	0 [0]	3 [7]	9 [16]	12 [7]
widowed	3 [5]	4 [9]	1 [1]	8 [5]
Employment status				
paid work	22 [36]	17 [37]	17 [30]	56 [34]
self-employed	11 [18]	8 [17]	4 [7]	23 [14]
non-paid work	2 [3]	1 [2]	5 [8]	8 [5]
unemployed	4 [7]	2 [4]	12 [21]	18 [18]
retired	1 [2]	1 [2]	0 [0]	2 [1]
student	1 [2]	1 [2]	7 [12]	9 [6]
others	1 [2]	1 [2]	0 [0]	2 [1]
No legal work permission	44 [72]	33 [71]	16 [28]	93 [57]

*Note: N*_*J*_ number of participants in Jordan, *N*_*T*_ number of participants in Turkey, *N*_*S*_ number of participants in Switzerland

### Overview reported problems

Across all countries, over half (59%) of participants stated two or three problems. Participants who stated more than one problem per question either described causes and consequences or listed multiple single problems. Between the countries, problem types were similar with identical categories but varied in frequency. As shown in Table 2, the main two categories were a) practical and b) psychological. Further frequent categories encompassed social issues such as interpersonal problems, separation from family members and problems related to family duties, and physical and psychosomatic problems.

**Table 2:** Overview categories of self-reported problems of the three study populations

Frequent problem category	Jordan	Turkey	Switzerland	Total
	N_J_ = 61	N_T_ = 46	N_S_ = 57	*N* = 164
	n [%N_J_]	n [%N_T_]	n [%N_S_]	n [%*N*]
Practical	44 [72]	31 [67]	39 [68]	114 [70]
Psychological	27 [44]	15 [33]	23 [40]	65 [40]
Interpersonal	15 [25]	14 [30]	15 [26]	44 [27]
Physical/psychosomatic health	13 [21]	13 [28]	10 [18]	36 [22]
Separation from family members	11 [18]	8 [17]	13 [23]	32 [20]
Related to war/home country	7 [11]	6 [14]	7 [12]	20 [12]
Related to family duties	10 [16]	5 [13]	2 [4]	17 [10]
Personal development/unmet personal needs	0 [0]	3 [7]	7 [12]	10 [6]

*Note: N*_*J*_ number of participants in Jordan, *N*_*T*_ number of participants in Turkey, *N*_*S*_ number of participants in Switzerland, *n/a* not applicable

### Practical problems

For a majority (*n* = 114, 70%) the troubling problems were of a practical nature. A prominent concern among all participants was employment, whereas camp-related issues emerged only in Jordan. Financial concerns were most prevalent in Turkey and government-related issues and housing were of greater concern in Switzerland. A further breakdown of the coding is reported in Table 3.

**Table 3:** Sub-codes of practical problem category of the three study populations

Sub-codes practical problems	Jordan	Turkey	Switzerland	Total
	n [%N_J_]	n [%N_T_]	n [%N_S_]	n [%*N*]
Employment	16 [26]	8 [17]	18 [32]	42 [26]
Finances	11 [18]	17 [37]	6 [11]	34 [21]
Camp related issues	26 [43]	n/a	n/a	26 [16]
Government regulations	3 [5]	4 [9]	12 [21]	19 [12]
Housing	2 [3]	5 [11]	11 [19]	18 [11]
Medical treatment	6 [10]	6 [13]	3 [5]	15 [9]
Education	2 [3]	5 [11]	6 [11]	13 [8]
Language	n/a	7 [15]	6 [11]	13 [8]
Others (e.g., food, climate)	1 [2]	0 [0]	2 [4]	3 [2]

*Note: n/a not applicable*

#### Jordan

Camp issues were identified by a significant proportion of participants in the Jordan sample (*n* = 26, 43%). Many participants expressed impairment of freedom, oppression and enforcement of being in the camp (*n* = 11), whereby eight directly linked those problems to Village 5, and challenging living conditions (*n* = 10), such as long waiting time for supplies, electricity shortage, distances to services or general terms for living conditions. Examples of the salient practical problems are shown in Table 4.

There were common concerns about employment (*n* = 16, 26%). Participants expressed causes such as governmental regulations on working permits, stay in the restricted Village 5 of the camp, lack of job opportunities, or financial stress associated with unemployment.

Financial concerns were the third most frequent practical problem (*n* = 11, 18%). Seven used general terms such as ‘financial problems’ or ‘lack of money’, whilst others related to house and childcare expenses, rising prices or were challenged by the role change to primary provider.

A slightly less prominent problem was not receiving appropriate medical treatment or worrying about it (*n* = 6, 10%). Residence permit issues assigned to government regulations, housing, education and climate issues were of minor importance.

#### Turkey

Many participants stated financial problems (*n* = 17, 37%) in Turkey. Most participants expressed financial issues in a general way (*n* = 9). They stated causes for the financial problems such as the lack of financial support by an institution/organization, no work or the associated consequences, such as not receiving appropriate medical treatment or financial instability.

Regarding employment problems (*n* = 8, 17%), four participants related these problems to financial stress. When considering work status, four people pursued paid work, three were homemakers and only one was unemployed. Respondents expressed problems such as not finding a job, followed by work disability due to health reasons, and the lack of social security after leaving a job. One participant expressed problems at work, including discrimination.

Challenge of learning a new language was described as a problem (*n* = 7, 15%), including impact on education and inability to learn Turkish due to family duties. Other less frequently stated problems were medical treatment (often because of financial limitations), unsuitable housing, children’s problems at school, and government regulations such as current residence permits preventing receipt of appropriate medical treatment or educational opportunity.

#### Switzerland

Eighteen participants (32%) expressed employment problems, whereby sixteen stated concerns about finding a job/inability to work, some being unable to find a suitable job according to their original profession or expectation. Furthermore, participants expressed difficulties in finding an apprenticeship or pursuing their studies or linked language competence with successful job seeking. One person referred to exploitation at work.

Eight out of twelve participants, who stated problems with government regulations, commented on the negative impact of residency status on freedom of movement, family reunion and temporary residency.

Another common problem was housing (*n* = 11, 19%), such as finding an appropriate apartment, dissatisfaction with living conditions in asylum centres or cost of rent.

Educational, financial and language problems were each mentioned by six (11%) participants. Beside general terms, specific descriptions ranged from finding an education place or apprenticeship, discrimination at school, cost of housing, financial uncertainty, and inconvenience in daily activities due lacking knowledge of the German language. Medical treatment (*n* = 3, 5%) and other problems (food) (*n* = 2, 4%) were very minor.

**Table 4:** Examples of top three practical problems

Jordan	Turkey	Switzerland
Employment	Employment	Employment
*“No job and can’t get a work permit” [J62]*	*“Can’t find work” [T18]*	*“To find a good job position. I am Arabic teacher” [CH34]*
*“[…] being unable to work (because I stay in village 5)” [J38]*	*“My husband is ill, I can’t work. We can’t have enough money” [T17]*	*“Difficulties with finding a job despite learning the language” [CH30]*
Finances	Finances	Government regulations
*“Financial problems / house and children’s expenses” [J33]*	*“I need an implant but I can’t due to financial problems” [T37]*	*“I feel temporary and not safe here because I have F […] my son also has F permit; he can’t travel out of Switzerland” [CH36]*
*“Being the family breadwinner because my husband is abroad” [J5]*	*“Unstable, I have no home or financial source” [T16]*	*“The immigration department rejected my asylum request to join my family” [CH2]*
Camp related	Language	Housing
*“Staying in the village 5 makes me feel unable to go out” [J38]*	*“My kids don’t know the Turkish language; they don’t understand their lessons” [T41]*	*“I don’t have an appropriate apartment for my children and me” [CH31]*
*“Being obliged to be in the camp bothers my children and I [...]” [J48]*	*“I can’t learn Turkish because I have many responsibilities, house and kids” [T20]*	*“Living in the asylum centre with the family” [CH13]*

Taken together, this section shows how strongly practical issues are linked to displacement-related factors, such as employment, government regulations, and language difficulties.

### Psychological problems

Various emotional, cognitive and behavioural symptoms of mental disorders such as depression, anxiety, posttraumatic stress disorder (PTSD), and psychotic symptoms were expressed in all three countries, illustrated in Table 5. Many issues were mentioned only once. In general, participants did not use diagnostic labels with one exception of ‘psychosis’ (a participant’s sibling). They used various terms of emotional or psychological symptoms (translated as e.g. ‘*anxious’* ‘*concentration problems’*), described their feelings by using simple language (‘*I feel bad..*’ ‘*I blame myself..*’) or used general terms (‘*mental health struggle’* ‘*emotional problems’*). In Jordan, frequent problems included worries about another person (*n* = 7, 11%), fear and anxiety (*n* = 6, 10%) and anger (*n* = 5, 8%) as was in Switzerland the feeling of uncertainty/insecurity (*n* = 9, 16%). The emotional impact of war, such as missing family members, memories of lost family members and past events in Syria, and fear for family members back in Syria, were of similar importance across all countries. In Turkey, the great number and variability of psychological problems endorsed by participants didn’t allow to build more particular problem categories. Psychological problems were frequently related to other non-psychological problems, either as causes or as consequences. One’s social network seemed to play a crucial role since over half of the psychological problems were of psychosocial nature meaning the interrelation of social environment and psychological wellbeing and hence, showed frequent overlap with social problems, others’ health problems, and the wellbeing of family members. Of 65 (40%) participants who mentioned psychological problems, 35 indicated also practical problems, with some making a causal link with psychological issues (e.g. causes such as language, financial, job difficulties).

**Table 5:** Examples of psychological problems

Jordan	Turkey	Switzerland
*“Worrying about my husband since he’s sick[…]*” *[J9]*	*“Feeling alone and have no friends” [T9]*	*“I don’t feel secure here because I am temporary and I have F” [CH36]*
*“I am constantly anxious” [J48]*	*“The illness of my daughter, I am afraid of losing her” [T15]*	*“I am worried because I turn 65 years and I don’t have enough money to live securely” [CH55]*
*“Being afraid to go back to my home country since we have no house left and no job” [J57]*	*“Remembering the bad time that we had” [T21]*	*“The situation in northern Syria […]. My father insists on staying in the city despite the war and I fear for them” [CH54]*

Overall, the results show the prevalence of psychological problems and the importance of social network and local displacement-related factors on psychological wellbeing, and illustrate psychological problems as the common consequence of a range of stressors.

### Social problems

Three categories could be assigned to social problems, which encompass issues usually resulting from a personal or societal relationship. First, interpersonal aspects (*n* = 44, 27%) consisted of interpersonal problems within the family such as conflicts or concerns with relationships with family members (Jordan = 10, 16%; Turkey = 9, 20%; Switzerland = 8, 14%). Another recurring interpersonal problem across all settings was undignified behaviour such as physical, verbal, or non-verbal violence towards individuals (Jordan = 7, 11%; Turkey = 5, 11%; Switzerland = 3, 5%). However, conflicts linked to the host community and displacement-related factors such as discrimination, labour exploitation, oppression were present only in Turkey and Switzerland. Second, separation from family members (Jordan = 11, 18%; Turkey = 8, 17%; Switzerland = 13, 23%) was commonly described as emotional and geographical separation, whereas the category of family reunion, meaning the inability of reunification of family members in the host country, emerged only in Switzerland (*n* = 5). Leaving/divorce from spouse assigned to interpersonal problems, as well as consequences of separation from family members, were expressed by participants in Turkey (*n* = 2) and Switzerland (*n* = 3). Third, family duties (Jordan = 10, 16%; Turkey = 5, 13%, Switzerland = 2, 4%), mostly described as household chores and parental responsibilities such as upbringing of, marrying off children and consequences of family dynamics, were more frequent among participants in Jordan and Turkey compared to Switzerland. Exemplar quotes of social problems are shown in Table 6.

**Table 6:** Examples of social problems

Jordan	Turkey	Switzerland
*“My daughter is often anxious; she hits her siblings” [J24]*	*“Difficulties to understand and deal with my kids, I blame myself because I treated them harshly […]” [T46]*	*“The loneliness I suffer since my wife has recently left me” [CH11]*
*“Being exploited by some people” [J45]*	*“The discrimination in the work place” [T19]*	*“Racism. I have two children and they don’t have the same treatment at school” [CH52]*
*“Inability to marry my son off in the Al-Azraq camp” [J49]*	*“My mom and my sister’s death” [T37]*	*“Not being able to reunite with my daughter” [CH30]*

This category highlights the role of stress within the familial social network, but also through the host society such as conflict, health-related, and displacement-related concerns within the family/community. Furthermore, the results also show a frequent overlap of interpersonal problems, separation from family members and family duties with psychological problems.

### Physical and psychosomatic problems

Across the three study sites a broad range of health issues was described and rarely mentioned more than once (e.g. eye problems, infections, stroke, and sleep). Additionally, participants often used general terms (e.g. physical problems, sickness) or cultural idioms (e.g. “*feeling weight on my chest*” meaning suffering from cancer). For participants in Jordan and Turkey, worries due to another person’s health problem (Jordan = 8, 13%, Turkey = 9, 19%, Switzerland = 3, 5%), as well as connection between health and practical problems, such as lack of appropriate treatment, and in Turkey, inability to work were more common. In Switzerland, possible psychosomatic symptoms such as pain (*n* = 5, 9%) were of relevant concern compared to Jordan and Turkey. Some of the above described results are exemplified in Table 7.

**Table 7:** Examples of physical and psychosomatic problems

Jordan	Turkey	Switzerland
*“Worried about my mother since she’s suffering from several health problems and her pressure is almost always high” [J26]*	*“The hard illness, that I had hepatitis B, the medicine is very expensive” [T4]*	*“Physical pain” [CH29]*
*“Children’s diseases (haemorrhage) and afraid not to find the necessary treatment” [J22]*	*“The illness of my daughter, I am afraid of losing her” [T15]*	*“Sleep […]” [CH3]*

These results indicate that not only personal physical integrity, but also health issues in the social milieu critically influence psychological wellbeing. Furthermore, health issues are associated with displacement-related factors.

### Personal development/unmet personal needs

There was a sense of impairment in personal development and meeting personal needs, including future job possibilities, having a family, or other aspirations which are illustrated by examples in Table 8. These problems occurred only in Turkey (*n* = 3, 7%) and Switzerland (*n* = 7, 12%).

**Table 8:** Examples of personal development/unmet personal needs

Turkey	Switzerland
*“I cannot complete my successful way, I stopped in the middle” [T38]*	*“I’m trying to achieve my objectives, but I need an opportunity” [CH32],*
*“Can’t do anything because of the kids” [T29]*	*“I can’t achieve my dreams in Switzerland” [CH41]*

## Discussion

This study presents the idiographic perspectives of distressed SRAs on their problems after their flight to Jordan, Turkey or Switzerland. To our knowledge, this is the first attempt to compare self-reported problems of a vulnerable group of SRAs between three host countries based on a standardized client-generated outcome measurement.

We found similarities and differences between the settings. In summary, the results show that SRAs across the three countries suffer from post-migration problems frequently related to their social ecology: primarily practical, followed by psychological, social, physical and psychosomatic problems. The findings differed somewhat between the settings, with camp-related and employment-related issues more common in Jordan, financial problems in Turkey, and employment, government regulations and housing problems in Switzerland. The finding of post-migration problems as major concerns of refugees and asylum seekers concurs with previous studies demonstrating the importance of post-migration stressors [6, 27, 49, 50].

Demographic characteristics and contextual factors in Jordan, Turkey and Switzerland differed in a number of important ways. It is possible that education affected the migration destination as the Swiss sample showed more highly educated SRAs than Jordan and Turkey. Similar to our results, Carlson & Williams [51] found a positive selection for advanced education in Syrian refugees migrating to Europe compared to Syrian refugees resettling in neighbouring countries, such as Lebanon or Jordan. A study by Brücker and colleagues [52] found a similar pattern in a sample of Syrian refugees in Germany. Previous research suggested that advantaged social positions relate to an earlier flight which could explain the tendency of an earlier flight in the Swiss sample [53]. In Turkey and Switzerland, the study design for recruitment implied a help-seeking behaviour, whereas in Jordan assessors conducted door-to-door screening. Therefore, it can be assumed that in Turkey and Switzerland participants may be more affected and hence, showed more interest in receiving help.

This study revealed that SRAs face overarching problems occurring in a similar way in all host settings as well as country-specific problems, more prevalent to the local setting. The overarching problems (psychological, social, physical and psychosomatic problems) are in line with previous findings [6, 22, 49], and hence, the similar aspects will not be discussed any further here. In contrast, great variation emerged within practical problems, which can be accounted for by country-specific displacement-related factors.

In terms of country-specific problems, camp-related concerns in Jordan such as physical safety concerns, unmet basic needs such as access to clean water, sanitation, medical services, adequate shelter, impairment of free movement, work exploitation and unmet psychosocial needs have also been highlighted in previous studies [11, 13, 30, 54]. Our study reflects the subjective burden of strong restrictions of freedom and limited access to work [41]. However, participants did not express concerns about physical safety, access to clean water, sanitation, work exploitation and shelter (caravan). A possible assumption that caravans in Al-Azraq camp were relatively adequate shelters is supported by a study, which found significantly greater dissatisfaction and need for psychological support in refugees staying in tents than in their counterparts staying in caravans [55]. However, a comparative study between Syrians in camp and non-camp-settings in Iraq found that refugees in camps are better off, particularly in terms of access to food, education, registration and employment [56].

Financial issues were the primary problem in Turkey, which may be associated with widespread informal work and low payment due to the temporary protection status [57]. Even though Turkey adopted the Regulation on Work Permits for Foreigners under Temporary Protection in 2016, access to the labour market remained challenging and exploitation and discrimination, also described in our study, as well as harassment occur frequently [19]. Our study supports the difficulty of meeting financial needs despite official working permits as half of those who cited financial problems owned an official working permit and half of the participants who reported an employment problem related it to financial problems.

A further displacement-related stressor, especially in Jordan and Switzerland, was employment, which is consistent with previous research [6, 58]. However, certain contributing factors and consequences may vary between the countries. In Switzerland, it seems possible that the high percentage of employment issues is associated with the high rate of unemployment and labour market restrictions for asylum seekers. Our study partly reflects labour market barriers found among refugees elsewhere, including visa restrictions, poor language skills, qualifications from their home countries not being recognised, discrimination, lack of vocational skills and psychological or physical barriers [59]. The complexity and far reaching consequences of employment difficulties were shown in a previous study illustrating the relationship between temporary visa status or discrimination affecting employment opportunities and thus, financial issues subsequently affecting the refugee’s social standing. Restricted employment opportunities may also affect affordable housing, which leads to increased instability, and in turn, to greater isolation [10]. In contrast, employment barriers in Jordan arguably can be attributed to sampling within a restricted camp that has less opportunity for employment.

In Switzerland, our findings show the strong impact of the immigration policy of temporary admission and long asylum seeking process on subjective wellbeing; this finding is in line with a previous study [60] and supports the potential of its change for mental health improvement [61]. Research outside of Switzerland has shown that temporary protection independently increased the risk of ongoing PTSD, mental-health related disability, mood and anxiety disorders and hampered socioeconomic integration [7, 29, 62]. It is assumed that the instability due to the temporary status also impacted the high percentage of participants with feelings of uncertainty/insecurity. This association and its implication for health and wellbeing has been established in a previous study [63]. Our results also shed light on the negative impact of living in an asylum centre in Switzerland, which supports the evidence of previous research in- and outside Switzerland, showing its detrimental effect on mental health and subsequent successful socioeconomic integration [18, 29, 64].

One further finding of the present study, to be mentioned is, that the host society has a distinct impact in community- vs. camp-setting on experienced problems: In Turkey and Switzerland participants reported undignified behaviour such as discrimination, racism by the host-society, which is in line with previous research [14, 18] and may strongly affect mental health, social adaptation, fosters internalising behaviour and hampers successful socioeconomic integration [10, 28, 65]. It can be assumed that the more interaction with the host-society the more undignified behaviour arises. However, in Jordan, feelings of marginalisation from national and international agencies were not reported as it was demonstrated by previous research [12]. On the other hand, personal development/unmet needs were only reported in the community-settings, e.g. Turkey, but not in the camp-setting Jordan. Whether restricted opportunities within the camp are accountable may be doubtful but the findings highlight that participants in Jordan are only worried about daily life circumstances, longing for family and life in Syria/elsewhere, or general future uncertainty. This may emphasise the hopelessness camp residents feel about the future and the “life after the camp” [55, 66].

### Limitations

This study has a number of limitations. The major weakness in this study is the transcription of the responses. PSYCHLOPS is designed for short responses. In Jordan, and partly in Switzerland as well, research assistants summarized the participants’ responses and hence, it is possible that participants’ own words were lost despite proper training in transcription. In Turkey, however, participants were instructed to share a synopsis, which would then be transcribed word-by-word. In both cases, the depth of the content of the actual experienced problems is reduced. More extensive responses would allow drawing a clearer picture of the participants’ perception and additional in-depth interviews could enable to investigate detailed causalities and consequences of certain problems. Considering the variability of transcription mode, it is suggested that stricter guidance of the interviewers on how to write down PSYCHLOPS responses is recommended. Additionally, the richness of the data might be reduced due to the Arabic-German translations in Switzerland and Arabic-English translations in Jordan, Turkey and Switzerland. Since the study is limited to three unique sites, and the sample populations were selected based upon a certain distress level, generalization to the entire populations in these and other countries is not possible. However, through the inclusion of distressed individuals the problems of a vulnerable group are highlighted and hence, can help tailor appropriate psychological and other interventions for this target group.

### Public health implications

Since only distressed SRAs as a particularly vulnerable population were included in this study, our findings could be of critical interest to immigration and public health policy makers as well as involved health and migrant organizations. Decision makers should be aware that practical stressors may have major consequences for psychological functioning and that all types of daily stressors may negatively impact successful integration [10, 61]. The alleviation of post-migration stressors is therefore critical not only for the individual wellbeing or ethical reasons, but also with regard to long-term socioeconomic aspects of hosting societies [10]. Therefore, authorities should carefully balance their interventions and consider the return on investment of appropriate health care und psychosocial support. In doing so, our findings suggest that target solutions should specifically address the site-specific needs of SRAs.

## Conclusion

This study was a first attempt to explore and compare the self-reported problems in SRAs resettled in three different countries in the Middle East and Europe. The results clearly indicate that distressed SRAs mostly suffer from a variety of post-migration stressors. Whereas psychological, social, physical and psychosomatic stressors are similarly distributed across study sites, practical problems show substantial variation between host countries and should therefore be specifically addressed by policy makers in order to foster psychological wellbeing and psychosocial integration.

Future research should explore the presented country-specific problems, underlying processes, causes and consequences in greater depth. Furthermore, since this study unilaterally examined problems, it is to highlight that a greater focus on resilience factors and empowerment could produce interesting findings that account for a more comprehensive needs analysis from refugees’ own perspectives.

## Supplementary Information

**Additional file 1: Table S1.** Supplemental examples of problems.

## Data Availability

The datasets are available upon reasonable request from the corresponding author.

## Abbreviations

SRAs: Syrian refugees and asylum seekers
PM+: Problem Management Plus
RASASA: Refugee and Asylum Seekers Assistance and Solidarity Association
PSYCHLOPS: Psychological Outcomes Profile
PTSD: Posttraumatic stress disorder
