# Supplementary material for: Problems after flight: understanding and comparing Syrians’ perspectives in the Middle East and Europe
Source: BMC Public Health. 2021 Apr 13;21:717. doi: 10.1186/s12889-021-10498-1 (PMC8045311; doi:10.1186/s12889-021-10498-1)
Supplement: Supplementary file 1 — Additional file 1: Table S1. Supplemental examples of problems. [file 12889_2021_10498_MOESM1_ESM.docx]

# Running Head: Syrian refugees’ problems after flight

# Problems After Flight: Understanding and Comparing Syrians’ Perspectives in the Middle East and Europe

Andrea Drescher^1,2^, Nikolai Kiselev^1^, Aemal Akhtar^3,5^, Ceren Acarturk^4^, Richard A. Bryant^3^, Zeynep Ilkkursun^4^, Roland von Känel^1,2^, Kenneth E. Miller^6^, Monique C. Pfaltz^1,2^, Matthis Schick^1,2^, Ulrich Schnyder^1^, Marit Sijbrandij^5^, Julia Spaaij^1^, Naser Morina^1,2^

and on behalf of the STRENGTHS Consortium

^1^ Department of Consultation-Liaison Psychiatry and Psychosomatic Medicine, University Hospital Zurich, University of Zurich, Culmannstrasse 8, 8091 Zurich, Switzerland

^2^Medical Faculty, University of Zurich, Zurich, Switzerland

^3^School of Psychology, University of New South Wales, Sydney, NSW 2052, Australia

^4^ Department of Psychology, Koc University, Istanbul, Turkey

^5^Department of Clinical, Neuro- and Developmental Psychology and WHO Collaborating Centre for Research and Dissemination of Psychological Interventions, Vrije Universiteit Amsterdam, The Netherlands

^6^ War Child Holland, Hemholtzstraat 61, 1098 LE Amsterdam, The Netherlands

Correspondence:

Naser Morina, PhD, Department of Consultation-Liaison Psychiatry and Psychosomatic Medicine, University Hospital Zurich, University of Zurich, Culmannstrasse 8, CH-8091 Zurich, Switzerland, naser.morina@usz.ch, Tel +41 44 255 52 80

Table 1

Supplemental Examples of problems

| Jordan | Turkey | Switzerland |
| --- | --- | --- |
| Examples of practical problems | | |
| Employment | Employment | Employment |
| *“No job and no money” [J56]* | “Not having salary after leaving job” [T5] | *“It really annoys me, the exploitation by the employers. That they only want some hands to work but don’t offer or pay” [CH7]* |
| *“Lack of job opportunities” [J16]* | *“Leaving Syria, can't find a job”* [T8] | *“Professional future: It hasn’t worked out with the job or the apprenticeship” [CH39]* |
| *“Work / trying to find a job for her son” [J61]* | *“Husband’s work circumstances and the financial issue. The husband is not working” [T33]* | *“I cannot work because I don’t get a good job. I don’t want to work as cleaning lady” [CH5]* |
| Finances | Finances | Finances |
| *“No money” [J39]* | *“No money” [4]* | *“The rent is not cheap” [CH33]* |
| *“Rising prices” [J50]* | *“Lack of money, because my husband has to send money to his family in Syria” [T14]* | *“I am worried because I turn 65 years and I don’t have enough money to live securely” [CH55]* |
| *“No money making me restless” [J43]* | *“There is no aid. I was having before, but they stopped it because my daughter became 18 years old - but I still have small kids” [T46]* | *“Finding a job with a decent salary” [CH38]* |
| Camp-related | Language | Language |
| *”Being in the Al-Azraq camp/want to go back to Za’atari” [J62]* | *“Learning Turkish language” [24]* | *“I have difficulties with Swiss German” [CH40]* |
| *“[…] Financial issues due to being unable to work (Because I stay in the 5th village)” [J38]* | *“The treatment my daughter receives because of the language barrier. I am also not able to explain this to the management” [T11]* | *“I have stress when I go to the gynecologist because I have no interpreter and I have to take my son with me” [CH53]* |
| *“Being at the camp while looking for better living conditions” [J10]* | *“Don’t know Turkish” [T1]* | *“Learning the language” [CH9]* |
| Government regulations | Government regulations | Government regulations |
| *“Visa expires before the end of this month. Duration of the work permit” [J28]* | *“My mother does not have a resident to do surgery” [T24]* | *“I have no passport; I have been here for three years and I still have the residence permit N” [CH50]* |
| *“Getting a working permit for my husband” [J29]* | *“I can't have a resident card till given that I have been in Turkey for 1 year and because of that my kids couldn't go to school” [T45]* | *“I don’t feel secure and here because I have a temporary residency, I have the F permit. My son has also F, he cannot travel out of Switzerland” [CH36]* |
| *“No job and can’t get a work permit” [J3]* | *“Can't get the ID” [T36]* | *“Residency, I still have the N permit” [CH17]* |
| Housing | Housing | Housing |
| *“Being at the camp while looking for better living conditions” [J10]* | *“My family has 14 members. We can't find a house that could fit us” [T12]* | *“I don’t find a house. I have to go back to the asylum centre” [CH57]* |
| *“Obligation to move from caravan and not being able to get the new caravan contract” [J29]* | *“Not being able of finding home” [T39]* | *“It bothers me living in the asylum centre with my family and I have difficulties finding an apartment” [CH13]* |
|  | *“My home is in 1st floor and its uncomfortable” [T40]* | *“I am in Switzerland since 8 months and I don’t have an apartment” [CH4]* |
| Education | Education | Education |
| *“My son Mohammad is illiterate” [J9]* | *“My son's problem at the school […]” [T44]* | *“[…] and not being able to work or study” [CH2]* |
| *“My son didn’t go back to school” [J40]* | *“I can't have a resident card till giving that I have been in Turkey for 1 year and because of that my kids couldn't go to school” [T45]* | *“Finding an apprenticehip” [CH46]* |
|  | *“[…] children education”*  *[T4]* | *“The difficulty of dealing with my children's supervisors in schools” [CH38]* |
| Medical treatment | Medical treatment | Medical treatment |
| *“Worrying about my husband since he’s sick, he suffers from a stroke and he’s not getting the necessary treatment” [J9]* | *“I have a disabled daughter. I can't take her to the doctor because the sessions are far away” [T12]* | *“No good medical treatment” [CH18]* |
| *“Unavailability of a treatment for my son’s disease in addition to some health issues that I suffer from” [J12]* | *“Medecine is very expensive” [T4]* | *“Doctors don't take my issue serious” [CH10]* |
| *“Inability to receive adequate treatment for my health issues and the difficulty to find one” [J48]* | *“I am sick too and need money for my medicine” [T7]* | *“Medical treatment“ [CH16]* |
| Examples of problems related to war/home country | | |
| *“Worried about my siblings because they’re still in Syria; fearing they’d get arrested” [J26]* | *“Being far from my country and my family, I feel I am strange here” [T20]* | *“Bad memories about Syria” [CH24]* |
| *Fear of going back to our house in Syria [J40]* | *“[…] remembering the bad time that we had” [T21]* | *“The situation in northern Syria; the way updates in the last couple of weeks” [CH54]* |
| Examples of psychological problems | | |
| *“Fearing for my child when send him out to run errands for the house because of his extreme vision deficit” [J18]* | *“Difficulties to understand and deal with my kids, I blame myself because I treated them harshly but I don't know why. Maybe because of the fear that I have and the effect of the war” [T46]* | *“I am worried about my future in Switzerland especially because I am old (35y) and therefore, I am depressed. Sometimes, I loose my hope in life” [CH43]* |
| *“Mental health struggle due to circumstances” [J47]* | *“I am sad because my husband is stressed” [T41]* | *“No self-confidence” [CH14]* |
| *“I get mad at my son when he fights with the neighboring kids” [J21]* | *“I feel very upset because there is no one who understands me and I feel lonely” [T42]* | *“I am not a positive person and I am sometimes pessimistic even though I will finish my studies after the next semester and can find a good job” [CH40]* |
| Examples of social problems | | |
| Interpersonal Problems | | |
| *“One of the relatives hurts me and he’s a close one” [J13]* | *“The way that the Turkish society is dealing with me and with my kids in school” [T17]* | *“The divorce and when my ex-wife requests for the children after she has officially left them with me” [CH31]* |
| *“My problem with my husband is that he wants to go to Syria but I don’t want to because his family causes me headaches” [J55]* | *“I feel that people are trying to avoid me” [T32]* | *“The relationship with the refugees and my family” [CH15]* |
| Separation from family members | | |
| *“Missing my siblings and my children who aren’t in the camp” [J3]* | *“Being far from my country and my family[…]” [T20]* | *“Without my family” [CH44]* |
| *“Being separated from my family” [J11]* | *“My husband got married to another woman and then he travelled to Ethiopia for work, which makes me ask for divorce” [T35]* | *“Being far from my children who are currently in Lebanon, Syria and Netherlands, especially my daughter in Lebanon” [CH23]* |
| Family related problems | | |
| *“Not being able to provide comfort for them [children] and the inability to do so and take decent decisions bothers me and makes me feel sick of all the responsibility” [J48]* | *“Difficulty in controlling children” [T40]* | *“[…] Stress of familial responsibilities especially that I am the breadwinner working to better our conditions (education, work, finding a new house)” [CH49]* |
| *“Dealing with my children issues especially the ones that need my husband’s input” [J4]* | *“I can't learn Turkish because I have many responsibilities, house and kids” [T20]* | *“The divorce and when my ex-wife claims for the children after she has legally left them with me”*  *[CH31]* |
| Examples of physical and psychosomatic problems | | |
| *“My daughter is very sick and I can’t get to her” [J55]* | *“I am sick too and need money for my medicine” [T7]* | *“Headache” [CH8]* |
| *“Unavailability of a treatment for my son’s disease in addition to some health issues that I suffer from” [J12]* | *“My father was dead and two of my brothers are disabled and my wife is ill” [T25]* | *“My husband is sick” [CH47]* |
